# Fraud and misrepresentation in retail forest products exceeds U.S. forensic wood science capacity

**DOI:** 10.1371/journal.pone.0219917

**Published:** 2019-07-25

**Authors:** Alex C. Wiedenhoeft, John Simeone, Amy Smith, Meaghan Parker-Forney, Richard Soares, Akiva Fishman

**Affiliations:** 1 Center for Wood Anatomy Research, Forest Products Laboratory, Madison, WI, United States of America; 2 Department of Botany, University of Wisconsin, Madison, WI, United States of America; 3 Department of Forestry and Natural Resources, Purdue University, West Lafayette, IN, United States of America; 4 Ciências Biológicas (Botânica), Universidade Estadual Paulista–Botucatu, São Paulo, Brasil; 5 Simeone Consulting, LLC, Anchorage, AK, United States of America; 6 World Wildlife Fund, Washington, DC, United States of America; 7 World Resources Institute, Washington, DC, United States of America; Universidade Federal de Pernambuco, BRAZIL

## Abstract

Fraud and misrepresentation in forest products supply chains is often associated with illegal logging, but the extent of fraud in the U.S. forest products market, and the availability of forensic expertise to detect it, is unknown. We used forensic wood anatomy to test 183 specimens from 73 consumer products acquired from major U.S. retailers, surveyed U.S. experts regarding their forensic wood anatomy capacity, and conducted a proficiency-testing program of those experts. 62% of tested products (45 of 73) had one or more type of fraudulent or misrepresented claim. Survey respondents reported a total capacity of 830 wood specimens per year, and participants’ identification accuracy ranged from 6% to 92%. Given the extent of fraud and misrepresentation, U.S. wood forensic wood anatomy capacity does not scale with the need for such expertise. We call for increased training in forensic wood anatomy and its broader application in forest products supply chains to eliminate fraud and combat illegal logging.

## Introduction

The rate of deforestation and illegal logging in key timber producing regions is unsustainable, is a critical threat to global biodiversity and forest ecosystems, is implicated in funding armed conflicts, reduces funds available to producer governments in taxes, and is a major facet of transnational organized crime [1,2]. Wood products made from illegally logged timber depress prices for legal products, further driving the global timber market toward unmanaged and illegal harvest. The United States (U.S.) is by far the world’s largest importer of wood and wooden furniture by value (51.5 billion USD in 2017, representing 22% of all global imports), with the second largest importer, China, importing half of the U.S.’ value (25.7 billion USD in 2017, or 11% of global imports; [3]). Understanding its role in leading the global demand for consumer wood products, the U.S. adopted and amended two primary legal systems governing product claims in forest products, especially wood and wood-derived materials; CITES (Convention on International Trade in Endangered Species of Wild Fauna and Flora; 27 U.S.T. § 1087) and the Lacey Act (18 U.S.C. § 42–43; 16 U.S.C. § 3371–3378). Both CITES and the Lacey Act require importers to make affirmative declarations of the botanical identity and geographic origin of imported wood. Companies or consumers interested in trying to mitigate the chance that their purchasing decisions support illegal logging can use tools that estimate risk, such as the Forest Legality Initiative’s Risk Tool [4].

**Fig 1:**
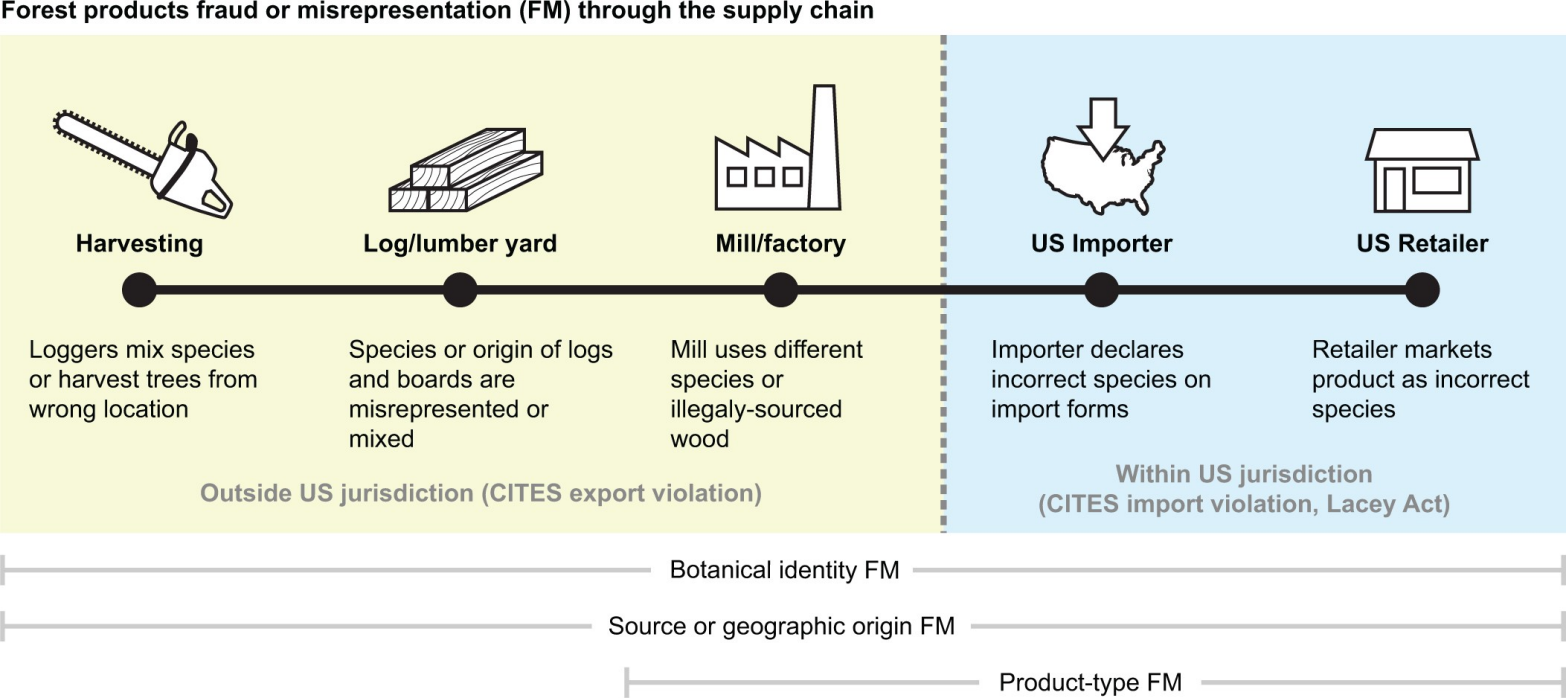
Types of forest products fraud and misrepresentation (FM), where they can occur in the supply chain, and jurisdictional boundaries for law enforcement. Botanical FM and origin FM can enter at any point, but product-type FM is necessarily a function of manufacturing, and so cannot enter the supply chain prior to that. The Lacey Act specifically depends on the source country defining the material as illegal, whereas CITES depends solely on the species or species and origin.

In addition to concerns about the legality of the raw materials used in forest products-derived consumer goods, there can be concerns about product misrepresentation or fraud in how products are described and sold. There are three primary types of fraud or misrepresentation (hereafter FM) in forest products, the botanical identity of the wood (e.g. the species), the source or geographic origin of the wood, and the product-type itself (e.g. solid wood vs. particleboard). The three different modes of FM can enter the forest products supply chain at different points, depending on the product and the type of FM (Fig 1) and can be intentional (fraud) or can be inferred to be the result of confusion or honest mistakes in species identification or supply chain management resulting in inaccurate product claims (misrepresentation). While it would be possible to use forensic wood anatomy at any step in the supply chain, most government inspections, if conducted at all, are made at the point of export from the country of origin, and/or at the point of import into the receiving country. Inspections or investigations are sometimes conducted by the mill/factory or the retailer, especially when a product differs from what was expected. Though there is little doubt that FM exists in the U.S. retail market for forest products derived consumer goods, the prevalence and severity of FM are as yet undocumented. Work related to fish and seafood supply chains demonstrates the utility of using forensic science to investigate natural resource supply chains in attempt to uncover the existence of fraud [5–7] and to improve traceability [8], but unlike seafood supply chains, there is no published scholarly data establishing the presence or scope of FM in forest products.

Forensic wood science approaches, such as DNA barcoding of wood [9–11] various types of chemical fingerprinting or chemometric approaches [12–14], or machine-vision wood identification [15–17] could be valuable in investigating forest products, but forensic wood anatomy–the scientific identification of wood based on its cellular anatomy—is the most widely applicable to wood material derived from a global supply chain, as it is the most fully developed and “street ready” of the existing techniques [18–20]. The primary limitations of forensic wood anatomy are its general inability to achieve species-level identification, instead typically being accurate to the genus level (e.g. *Handroanthus* sp., a species of ipê, rather than *Handroanthus serratifolia*), and its inability to determine the geographic origin of wood. Both these limitations are a function of the inherent variability of wood structure compared to the constraints imposed by the question. For the case of botanical identification, species of plants are typically defined by morphological features of external vegetative and reproductive structures, none of which are present in wood. Wood anatomy does not vary characteristically at the species level for most woods [20]. Determination of origin depends on the existence and analysis of one or more characteristics of the wood proven to vary with geographic location. In the absence of such features, determination of origin is not possible.

Despite these limitations, forensic wood anatomy in conjunction with wood technological understanding provides a broad base from which to determine if there is evidence for forest products FM. Then, if FM is present, it is sensible to ask if the U.S. has existing expertise and capacity to meet current demand for forensic work. For the future, does the U.S. have the needed resources and proficiency to train new experts at a rate that can scale with the potential demand?

We present the first scholarly data demonstrating the presence of FM in forest products supply chains. We use forensic wood anatomy to test 73 consumer products acquired from major national retailers to determine the presence of FM in wood-derived common household goods available in the U.S. market. We compared product identification results to retail claims revealed *post hoc* to determine the prevalence and types of FM. To evaluate national capacity for forensic wood anatomy, we surveyed putative wood identification experts regarding their self-reported wood forensic ability and administered a voluntary wood identification proficiency-testing program. We discuss the scale and scope of forensic demand, and call for broader application of forensic wood anatomy in forest products supply chains.

## Results

### Fraud and misrepresentation (FM) in U.S. consumer forest products

We investigated 73 consumer forest products acquired in the U.S. market from major retailers for the presence of FM. We emphasized products that a typical American family might purchase–products included furniture, kitchen implements, sporting equipment, musical instruments, hand tools, home improvement materials, and other durable household items. As described in Materials and Methods, we selected consumer products from major national retailers based on claims indicating the use of woods likely to be high-risk. We selected national retailers because they are assumed to have more extensive resources for supply chain management, and because products available from a national retailer are likely found throughout the country and thus reflect broadly available products. We also made a distinction between the number of products (73), the number of product components (125), and the number of woods (183) that make up the product components, but we present the synoptic results of FM evaluation at the product level in Table 1. This evaluation of FM at the whole-product level is in keeping with law enforcement norms–if the fretboard of a guitar is illegal, the entire guitar is considered illegal. Finer-scale results at the product component and individual wood specimen levels are reported in S1 File, and narrative descriptions for each product can be found in S2 File.

**Table 1:** Composite claim results table showing numbers of products with correct and incorrect botanical and product-type claims, and the proportions of all products of each class.

	Number of products			Proportion of all products		
	Correct product-type	Incorrect product-type	Total	Correct product-type	Incorrect product-type	Total
**Correct botanical claim**	28	5	33	0.38	0.07	0.45
**Incorrect botanical claim**	30	10	40	0.41	0.14	0.55
**Total**	58	15	73	0.79	0.21	1

#### Botanical FM

40 of the 73 (55%) products tested showed clear evidence of botanical FM, with only 33 products (45%) being entirely made of woods consistent with the claimed species. For botanical FM, material can be completely misrepresented, or it can be commingled with properly identified wood. Such FM can be the result of an honest mistake, for example two closely related species that can only be separated by floral characteristics may be impossible to identify at the time of harvest because the trees are not flowering. Approximately 20% of the botanical FM claims we found could plausibly be attributed to honest mistakes and could be construed as misrepresentation. Conversely, loggers may selectively harvest high-value protected species but document them as lower-value woods, or a manufacturer or retailer may represent a low-value wood as higher-value wood. These latter two cases are examples of unambiguous fraud, whereas the former is an instance where a legitimate case for good-faith confusion and misrepresentation can be made. In all cases, the product claim is at a minimum misrepresented. When closely related or other similar species are mixed, as in the former example, such misrepresentation is not likely to be detected by the consumer, and may not appreciably impact the performance of the product.

Table 2 presents summary data for the woods found in the products we investigated, grouped according to broad categories of wood type: hardwoods (98%) vs. softwoods (2%), domestic (17%) vs. exotic (83%) species, and temperate (17%) vs. tropical (83%) woods. Conventional wisdom in the U.S. forest products market is that domestically produced timber and the resultant forest products are low risk for illegality, and that the bulk of risk exposure in the U.S. market comes from imports. If the proportions in Table 2 are roughly representative for higher-risk products in the U.S. market, this further informs our ability to understand the relevance of existing U.S. forensic wood anatomy capacity, by suggesting an approximate scale for the need for forensic capacity.

**Table 2:** Broad characterization of woods found in the commercial product components according to three categories: Hardwood vs. softwood, domestic vs. exotic, and temperate vs. tropical.

	Hardwoods	Softwoods	Domestic	Exotic	Temperate	Tropical
**Number**	117	3	21	99	21	99
**Proportion**	0.98	0.02	0.17	0.83	0.17	0.83

#### Origin FM

Forensic wood anatomy only rarely provides information about the origin of wood, other than at the broadest geographic scale, for example, specifying a continent or broad region of origin based on the natural distribution of a given taxon. To address forensic questions of the origin of the wood in a product, we would also need specific origin claims to test, and these were generally unavailable at the retail level for the products we studied. For these reasons, we did not attempt to address questions of wood origin.

#### Product-type FM

Results for product-type FM at the product level are reported in Table 1. 21% of the product-type claims were inaccurate in the products we evaluated. This is consistent with field observations in retail settings, where the most common type of FM is the claim of solid wood when in fact the product-type is a veneer adhered to non-solid-wood substrate (Wiedenhoeft, personal observation). Product-type FM is entirely human driven, as the product-type is the result of primary and secondary manufacturing choices and is not inherently dependent on the species or origin of the wood. Despite its dependence entirely on human choices, product-type FM can have gradations in severity. For example, plywood is not considered solid wood, but a maple butcher-block table top formed of many finger-jointed, glued-up pieces of solid wood is construed as solid wood in our data. If a consumer were expecting a single piece of wood, a product claim that called such a table top solid wood could be considered an honest mistake or misunderstanding. The same table top claiming solid wood construction, but made with a veneer of maple glued to a medium density fiberboard substrate is not solid wood, and would be a clear case of product-type FM.

#### Hybrid FM (botanical x product-type)

There are four domains of results when considering both botanical and product-type results: correct species and correct product-type, correct species and incorrect product-type, incorrect species and correct product-type, and incorrect species and incorrect product-type, shown in Table 1. The correct species and an incorrect product-type comprises the least common class (7% of products), followed by incorrect species and incorrect product-type (14%), followed in turn by products with the correct product-type and the correct species (38%), with the most common class being products with the correct product-type claim, but an incorrect species claim (41%). Only 28 of the 73 products sampled here were without some form of FM. Products without product-type FM are approximately equally likely to have a correct species claim as an incorrect one, but products with product-type FM are twice as likely to have an incorrect species claim as a correct one, though whether this is a result of intentional mislabeling or poor supply chain control cannot be determined from our data.

### Forensic capacity

#### Self-reported laboratory capacity

Complete survey responses are available in S3 File, with individual respondents’ names removed (the unredacted list is maintained by the editor and the authors); aggregated data are presented in the following text. The overall survey response rate (calculated using response rate definition one of [21]) was 53% (23 respondents of 43 survey recipients), which is approximately in keeping with trends in survey response rates near 52% [22]. 15 of 23 respondents reported some ability to engage in wood identification, but of those only 13 reported in detail about identification capacity. These 13 respondents identify approximately 830 specimens per year, of which about 25 specimens are unidentifiable. Most respondents report limited or absent ability to identify exotic and/or tropical woods, with only three respondents reporting global scope of their identification prowess. Of the 23 survey respondents, 9 (39%) agreed to participate in the proficiency testing, and of those 5 (55%) reported results.

For those who charge per specimen for identification, the cost per specimen ranges from 50 to 200 USD, with a turn-around time from a few days to a few weeks. Based on an average fee of approximately 65 USD per specimen, the total reported annual value of the identification work is roughly 54,000 USD at the current pace. If we assume that the respondents could roughly triple their annual work on forensic wood identification, the annual U.S. national forensic capacity outside the U.S. Forest Service, Forest Products Laboratory’s Center for Wood Anatomy Research would be in the vicinity of 2,500 specimens. Given that a minority of respondents profess ability to identify exotics but exotics are on average the higher-risk products, this 2,500 specimen total might sensibly be thought of as a best-case scenario for national capacity.

Our evaluation of 73 consumer products required 183 separate wood identifications. If we assume that this rate is broadly applicable across all wooden products in the U.S. market, 2,500 identifications amount to the capacity to fully investigate approximately 1,000 individual products per year. Even if all of this capacity were directed at verifying the claims of wood products in the retail market, given the U.S.’ position as the world’s largest importer of wood and wooden furniture, it is hard to imagine that this low number of product identifications is sufficient to deter forest products FM or turn the tide against illegal logging. To illustrate the point, the ability to conduct identifications on 1,000 individual products per year amounts to a sampling rate, just for wooden furniture imports, of one thousandth of a percent (0.001%) given that that the U.S. imported over 93 million individual wooden furniture items in 2017, valued at 18.5 billion USD, which was just under half the world’s total imports of wooden furniture. The majority of U.S. imports of wooden furniture are exported from China and Vietnam (47.7% and 20.0%, respectively, in 2017; [3]), and both countries commonly process woods from forests around the world, indicating an essentially global possible scope for wooden furniture in the U.S. market. These figures are only for wooden furniture and such concerns about FM are not restricted to furniture, but include U.S. imports of other wood products like flooring, plywood, musical instruments, sports equipment, or other wooden specialty products. Existing forensic capacity can thus be construed to be far less than 0.001% of the annual imports into the U.S.

#### Training capacity

Three respondents reported capacity to train additional personnel to a forensic level of expertise: one consultant reported an annual capacity of 30 people per year for domestic wood species and another consultant reported a capacity to train 50 people per year, presumably with a global purview for species. One academic reported the capacity to train one person per year, for an aggregate capacity of 81 individuals per year. These numbers presume training space, materials, salary, travel expenses, and though it was not stated by the respondents, such training would consume a major portion of each year by the consultants and trainees–it would likely require a minimum of 20 hours per week for a year to achieve reliable forensic proficiency for a core set of relevant commercial woods (Wiedenhoeft, personal estimation).

It is logical that if a respondent cannot accurately identify wood, it would not be valuable for them to train others in wood identification, but the converse is not necessarily true—ability to identify wood does not automatically imply the ability to impart or transfer that knowledge to others. Even though respondents reported a capacity to train 81 individuals per year, our survey did not assess respondents’ training ability.

#### Proficiency testing

Five laboratories participated in the proficiency testing–two from academia, and three consultants. The complete proficiency testing results are available in S4 File, but summary data regarding the performance of the five participants are noted in Table 3. As few as 10 specimens were attempted by one of the academics, and only one of the consultants attempted to identify every specimen in the set. Overall accuracy, evaluated at the genus level, based on the full set of specimens ranged from 6% to 76%. When accuracy was calculated, again at the genus level, for only those specimens that were attempted, the range shifted from 14% to 90%. All participants were 100% accurate identifying whether each specimen was a hardwood or a softwood. If we exclude participant 1 for having attempted only 10 of 55 specimens and then calculate average performance values using only participants 2–5, we see that on average participants attempted to identify twice as many domestic as exotic specimens, and more than three times as many temperate as tropical woods (Table 3). Regardless of whether we code the woods as domestic vs. exotic or temperate vs. tropical, we see similar average performance: roughly 80% accuracy for domestic/temperate woods, and roughly 50% accuracy for exotic/tropical woods. It is important to remember, of course, that these metrics are best-case scenarios where participants were allowed to omit specimens that they knew they could not identify–this is not possible when confronting a forensic specimen and the performance metric would be substantially lower. These average metrics are in keeping with conventional wisdom about domestic bias in U.S. forensic wood anatomy capacity.

**Table 3:** Proficiency testing results.

	Overall accuracy	Number of specimens attempted (number of specimens per class)				Proportion correct of those attempted (number of specimens per class)			
Participant	Full kit (55)	Domestic (28)	Exotic (27)	Temperate (32)	Tropical (23)	Domestic (28)	Exotic (27)	Temperate (32)	Tropical (23)
**1**	0.06	8	2	8	2	0.25	0.50	0.25	0.50
**2**	0.45	23	7	27	3	0.91	0.57	0.85	0.67
**3**	0.53	26	9	30	5	0.92	0.56	0.90	0.50
**4**	0.76	28	27	32	23	0.86	0.67	0.84	0.65
**5**	0.33	27	11	31	7	0.56	0.27	0.55	0.14
**Average of 2–5**	0.52	26	13.5	30	9.5	0.81	0.52	0.79	0.49

The overall accuracy for the full kit, then number of specimens attempted and the proportions correctly identified in the proficiency testing for the five participants. The metrics are partitioned according to the origin of the specimens, Domestic vs. Exotic and Temperate vs. Tropical. Participants are identified by a number code: 1 and 2 are from academia, 3 4, and 5 are consultants.

If we extrapolate these accuracy numbers backward to our estimate of total domestic forensic capacity of 2,500 specimens per year, and if we generously assume that 50% of woods are domestic and 50% are exotic, and we further use the success rates based on experts omitting specimens they know they cannot identify, for domestic woods we would have 88 unattempted, 221 incorrectly identified, and 942 correctly identified specimens. For exotic woods we would have 625 unattempted, 300 incorrectly identified, and 325 correctly identified specimens. This would be, essentially, a best-case scenario, with an aggregate success rate of just under 71% of the specimens attempted, or just under 51% of the total specimens. As the relative proportion of exotic woods increases the success rate decreases.

## Discussion

We provided the first-known scholarly evidence of botanical and product-type fraud and misrepresentation (FM) in the U.S. retail forest products market based on a broad sampling of common household items for sale from major national retailers. Such FM could be the result of upstream misdeclarations (e.g. illegally logged and illegally imported wood), mixing of multiple wood species that look alike during the manufacturing process, negligent or misguided efforts to market products accurately, or with intent to defraud the customer (Fig 1). As described in the Materials and Methods section, the range of products we surveyed included the kinds and costs of products that a normal American household might use, purchased from widely-recognized companies of sufficient size to be able to afford the infrastructure to perform due diligence in sourcing and marketing their products. Our sampling non-randomly targeted high-risk product claims and focused on finished consumer products, not logs, bulk products, timbers, or unfinished wood, although these other types of wood and wood products and the related sectors of the forest products economy from which they are derived might benefit from greater scrutiny as well. To accurately judge the true frequency of FM in the wood and wood products sector in the U.S. market would require a randomized study of such scope, scale, and cost that it would not be realistic to call for such an endeavor, especially if the results were to be anything other than a single snapshot in time. Because our approach was targeted and not randomized, we reiterate that our results–especially the overall frequency of FM–should not be extrapolated to the U.S. market more generally, but we are confident that our results correctly indicate the non-trivial presence of FM at large. This is also consistent with the observations of forest products in a wide range of retail settings over the last two decades, including the time since the implementation of the Lacey Act (Wiedenhoeft, personal observation). If a consumer had concerns about the responsible sourcing of their wood or wood-based products, one recourse would be to purchase certified products (e.g. Forest Stewardship Council, Sustainable Forestry Initiative, Programme for the Endorsement of Forest Certification, Rainforest Alliance Certified, others), although as yet we have no scholarly data to support the reliability of botanical claims or product-type claims in products from these systems.

By extrapolating the results of our capacity survey and proficiency testing results we also demonstrated a limited national forensic capacity. Overall accuracy for domestic wood species is near or below 90%, and sometimes far below, depending on how the metrics are calculated. Such an accuracy rate would be admirable for law enforcement officers screening products at ports or border crossings, but for forensic level laboratory analysis, it is insufficient. With an accuracy well below 60% for exotic woods, existing, generalized capacity is grossly inadequate. Given the comparative lack of ability to correctly identify unknown woods, it is not realistic to expect to train our way out of this lack of capacity, as the potential trainers themselves do not perform reliably at a forensic level. The short- and medium-term forecast, then, is a general lack of forensic capacity to support industrial compliance or law enforcement at a relevant, commercial scale, with limited prospects for improving this capacity without significant investment of resources in forensic wood anatomy training programs at institutions with sufficient expertise.

Our results for the U.S. market are the first published data for FM in forest products supply chains, and represent a first glimpse of a problem that is likely global in scope. Specifically, if our results for the U.S. market are roughly typical for net-consumer nations, especially for those with similar or less stringent legal mechanisms for preventing trade in illegal wood or consumer fraud, it is reasonable to conclude that forest product FM is a problem with global scope. Depending on where in the supply chain (Fig 1) faulty claims enter the system, the presence of FM could be indicative of illegal logging and supply chain mismanagement in producer countries as well, but to resolve this it would be necessary to apply forensic wood anatomy across the supply chain. If our results indicating a relative lack of capacity can be appropriately generalized to other countries, it would strongly suggest a global dearth of forensic wood anatomy capacity compared to the implied need for such expertise. In the absence of actions to increase capacity for industrial compliance with and governmental enforcement of laws governing forestry and trade, especially international trade, in forest products it would be prudent to assume that forest products FM will continue.

## Materials and methods

### Fraud and misrepresentation detection in retail forest products

#### Materials

Retailers were selected from Furniture/Today’s “2015 Top 100 U.S. Furniture Stores” [23] and the National Retail Federation’s “Top 100 Retailers 2015” [24]. The top 50 companies from each list were sorted to remove those that did not appear to sell solid wood products, resulting in an initial list of 46 possible companies representing the top furniture retailers and companies that sell wood products in the U.S. Seventy-three consumer products were purchased from 29 of these retailers, with mean and median number of products purchased per retailer being three and two, respectively. As an indicator of market relevance, the 29 retailers had combined 2014 U.S. sales of 829 billion USD [24]. Product costs ranged from less than 3 USD to over 900 USD, with an average per-product cost of approximately 154 USD. Consumer products marketed as either being exclusively or in part made from “high-risk” wood species (defined below) were selected for purchase. Potential product purchases were scrutinized to confirm that items chosen were those with affirmative wood species claims. Close attention was paid to the possibility of retailers using industry-acceptable trade names or specific species names in reference to surface finish (e.g. stain) coloring, or to provide a descriptive flourish for the item, but that stopped short of claiming to be made from that specific wood species. Products with these types of descriptions or claims were immediately rejected from consideration. In this way we targeted 73 products that were certain to contain affirmative claims as to the wood species utilized. With regard to the product claim, the term “species” is used to refer to the botanical wood type but may in fact refer to a genus or other taxonomic level. Because product claims were mostly made via common or trade names in product literature, we interpreted the claimed “species” on the basis of commonly or widely applied trade names (e.g. in the Center for Wood Anatomy Research’s common name database) [25].

Products were composed of one or more product components (each component given a separate designation, e.g. WWF-49A, WWF-49B, where these two components are different pieces from the same parent product), each of which were in turn composed of one or more woods (e.g. if a component were a plywood core and that plywood had 5 plies, there would be 5 separate identifications for that one component of the product). Results are generally reported by product or product component and overall product characterizations follow the bad-apple ethos—if at least one wood in at least one component is misrepresented, the entire parent product is coded as misrepresented. This is consistent with law enforcement norms at the scale of individual products.

#### Definition of wood products

We used standard trade data nomenclature (globally–Harmonized System codes- and in the U.S.–Harmonized Tariff Schedule)- “wood” is broadly understood as all products classified under Chapter 44 and “wooden furniture” is all commodities classified under the following 6-digit codes within Chapter 94: 940161, 940169, 940330, 940340, 940350, 940360.

#### Risk assignment

A database of high-risk wood species was assembled comprising those species previously identified by WWF as “high-risk” [26], which contained typically used common names, scientific name, countries of origin/harvest, and countries typically involved in trade. Additional species not named in the “WWF Country Profiles 2015” publication were taken from [27] and were added to the database of what was considered a “high-risk” species. In summary, 26 “high risk” species were represented as product or component species claims for the 73 products purchased.

#### Forensic analysis

Products were processed into multiple smaller test specimens, depending on whether the product contained multiple components or wood types, then labeled and submitted without any attendant product claim information for forensic analysis. Specimens were designated as WWF-##, and were tracked in a Google Docs spreadsheet. None of the above information about the logic of product selection was shared with the forensic team until after all laboratory results had been finalized.

Macroscopic observations of wood specimens were made with a 14X hand lens after cutting the transverse surface of the wood with a sharp knife to produce a transverse surface showing the cellular details of the wood. Observations made at this scale were functionally qualitative, and relied on expert knowledge of wood patterns rather than measured data or a list of explicit character states. Slides for microscopic analysis were prepared as in [28].

UV fluorescence of dry, solid wood surfaces and of water and ethanol extracts were used as supporting characters for targeted identification questions (e.g. fluorescence is not relevant or valuable in separating *Swietenia* from *Cedrela* but is useful in separating *Robinia* from *Morus*.) For surface fluorescence, fresh wood was exposed with a sharp knife and illuminated with a ~365 nm peak emission UV light source in a darkened room. For water and ethanol extracts, small shavings of cleanly cut wood were placed in ~5mL of deionized water or 95% ethanol in a clear glass cuvette and shaken, then illuminated as for solid wood. Observations for extract fluorescence (color, intensity, timing of extraction of fluorescent compounds in water or ethanol) were made over the course of approximately 5 minutes.

Observation of cells and cell features, whether via macroscopy, microscopy, or both, and comparison of those patterns and features to information in published keys, online databases (e.g. [29]), and ultimately to specimens in the MADw-SJRw xylarium housed in the Center for Wood Anatomy Research at the Forest Products Laboratory in Madison, WI, USA was the backbone of the identification process. In specific cases other features like fluorescence, odor, color, or reactivity (or lack thereof) to specific chemical reagents (e.g. Chrome Azurol-S) also informed the identification.

#### Classification of product claims

Each product had associated product claim information with varying degrees of specificity regarding both the botanical nature of the wood in the product (e.g. American black cherry effectively specifies a single species), and the type of product itself (e.g. solid wood table-top). We categorized the botanical claims according to an assessment of the product claim (e.g. “oak” is widely understood to be *Quercus* and only *Quercus*, whereas “rosewood” is less clear–without a modifier, “rosewood” is understood to be *Dalbergia*, but a name like “tiete rosewood” is species-suggestive of a different genus, *Guibourtia*, specifically one species in that genus *G*. *hymenaeifolia*). In cases where one portion of the product documentation claimed one wood and another portion of documentation claimed another, we assigned the presumed botanical identification based on whatever portion of the documentation was most visible to the consumer. For example, in WWF-20, a chair, the online product name and specifications list balau as the wood, which it is not. The assembly instructions clearly state that is it *acacia*, which is correct. Because the product claim information available at the time of purchase indicates balau, the product is coded as misrepresented (but such cases were neither typical of nor common in our data set).

Other product claims pertaining to the product type (construction or physical nature of the product) were evaluated according to common-use definitions and common-sense boundaries. For example, “solid wood” would include a board made of a single piece of wood and also a glued-up block or board (as with a butcher-block or as could be true of a table leg) where each piece is itself “solid wood” even though a lay person may not necessarily understand such a product to be considered solid wood. Excluded from this group would be any plywood product, as that is most commonly understood to be a separate category (plywood) where each component is a ply or veneer, not solid wood. A veneer or wear-layer adhered to MDF, to plywood, or to a particleboard core is not a solid wood product. A high-value veneer glued to a low-value solid stock wood was not “solid wood” of the high value species, but was considered “solid wood” more broadly.

#### Product claim communication and integrating forensic results

After provisional forensic identifications were posted, the shared spreadsheet was updated to indicate the product claims. Provisional identification results were compared to the product claim to determine if the context of the claim could inform the interpretation of the identification results. If product claim information influenced the final identification (which happened in five of 125 product components distributed over three of 73 products) it was noted.

### Domestic capacity survey and proficiency testing

Surveys were administered electronically (hosted on surveymonkey.com) or by interview, and the questions and recipients are found in S3 File. Outreach for survey completion was conducted primarily via email, and also by phone and limited in-person interviews. Survey data are self-reported and as such must be interpreted in that context. Prior experience with some of the respondents suggested that the self-reported ability may exceed actual ability. The paucity of respondents precludes most meaningful statistical hypothesis testing, but descriptive statistics are presented, calculated in Microsoft Excel.

Specimens for the wood identification proficiency testing were taken from the exchange collections of the MADw and SJRw xylaria at the Forest Products Laboratory, and were cut to various sizes such that all specimens of a given wood for each kit were equivalent, depending on the size of the parent block of wood. Each individual specimen was assigned a unique identification number so that individual specimens were trackable, and such that it would be effectively impossible for participants to compare results cooperatively. S4 File lists the species and numbers of specimens sent in each kit to the participants, as well as the participants’ results. Multiple entries for a species indicate different parent specimens.

Individual labs participating in the proficiency testing communicated their methods as part of the survey component of the study. Participants were asked to communicate their results as they would to a client or customer–we gave no appreciable guidance in this respect, for example, whether to use scientific or trade names, but merely provided a blank spreadsheet in which results could be recorded. Results were compared to the actual identification–accuracy is tabulated at the genus level, which is compatible with the scientific limits of forensic wood anatomy [20]–performance metrics would be much lower if calculated at the species level.

## Supporting information

S1 File Detailed product claim information and identification results, vendors redacted. (XLSX)

S2 File Product descriptions and detailed comments on product claim results, product-specific details redacted. (PDF)

S3 File Full responses to forensic capacity survey, identifying details redacted. (XLSX)

S4 File Proficiency testing results and summary metrics, identifying details redacted. (XLSX)
