# Supplementary material for: Fraud and misrepresentation in retail forest products exceeds U.S. forensic wood science capacity
Source: PLoS One. 2019 Jul 25;14(7):e0219917. doi: 10.1371/journal.pone.0219917 (PMC6657862; doi:10.1371/journal.pone.0219917)
Supplement: S2 File — (PDF) [file pone.0219917.s002.pdf]

Product-by-product testing results and comments.

Each of the product narratives that follow begin with the product number within the study and short product description with a product description materials provided by the retailer (retaining critical product claim information, but not using exact text so that the manufacturer cannot be easily searched for), then are followed by a description of the expected scientific name based on the product description along with any caveats associated with that claim. The actual wood identifications are reported next, with any additional information about the interface between uncertainty in the claim and inherent uncertainty in the identification.

WWF-WA1 Kitchen item made from a block of Chinese oak:

Chinese oak is presumed to be a species of *Quercus*, but could also potentially include *Pasania* and *Cyclobalanopsis*. Wood anatomy recognizes three groups of species within *Quercus*, the red oak group, the white oak group, and the live oak group.

The specimens from this product were identified as: *Fraxinus*. This is not consistent with the species claim. *Fraxinus* is a ring-porous hardwood like *Quercus* but is easily distinguished.

WWF-WA2 Mahogany wood handle:

Mahogany is presumed to be a species of *Swietenia*, unless the name is preceded by some other modifier (e.g. African mahogany, which is presumed to be *Khaya*, or Philippine mahogany, which is presumed to be *Shorea*.) All three accepted species of *Swietenia* are CITES Appendix II species, meaning that any product made from *Swietenia* should reasonably be traceable back to a valid CITES export/import permit. The presence of CITES-controlled species in either Appendix II or Appendix III in a wood product should probably be considered at least a yellow flag. Any CITES Appendix I wood is fully prohibited from trade and is an automatic red flag. There is also a claim of figured wood. Addressing the vagueness associated with the use of the word 'figure' is beyond the scope of this report, but it is important to note that no evaluation of the presence or absence of figure was made in this study.

The specimens from this product were identified as: *Entandrophragma* cf. *cylindricum* or sapele, from Africa. This is not consistent with the species claim. While sapele and mahogany are in the same botanical family they are in different genera and a number of features separate them.

WWF-WA3 Chair made from rubberwood:

Rubberwood is presumed to be *Hevea brasiliensis*. From a wood technology perspective, *Hevea* would not normally be considered among the set of "densely grained and sturdy" woods, nor would it be considered light, soft, or weak.

The specimens from this product were identified as: *Acacia* cf. *confusa* and *Hevea* cf. *brasiliensis*. Because *Acacia* was found, this is not consistent with the species claim - *Acacia* cf. *confusa* and *Hevea brasiliensis* are not confusable. Of note, however, is that both species are common plantation species in Asia.

WWF-WA4 Mahogany table:

Mahogany is presumed to be a species of *Swietenia*, unless the name is preceded by some other modifier (e.g. African mahogany, which is presumed to be *Khaya*, or Philippine mahogany, which is presumed to be *Shorea*.) All three accepted species of *Swietenia* are CITES Appendix II species, meaning that any product made from *Swietenia* should reasonably be traceable back to a valid CITES export/import permit. The presence of CITES-controlled species in either Appendix II or Appendix III in a wood product should probably be considered at least a yellow flag. Any CITES Appendix I wood is fully prohibited from trade and is an automatic red flag.

The specimens from this product were identified as: *Swietenia* sp. This is consistent with the species claim.

WWF-WA5 Rosewood handle:

Rosewood is presumed to refer to any species of *Dalbergia*, unless the name is preceded by some other modifier (e.g. tiete rosewood, which is presumed to be *Guibourtia chodatiana*). All species of *Dalbergia* are now CITES Appendix II species, meaning that any product made from *Dalbergia* should reasonably be traceable back to a valid CITES export/import permit. The presence of CITES-controlled species in either Appendix II or Appendix III in a wood product should probably be considered at least a yellow flag. Any CITES Appendix I wood is fully prohibited from trade and is an automatic red flag.

The specimens from this product were identified as: *Guibourtia* cf. *tessmannii*. This is not consistent with the species claim, and further would likely represent a CITES violation.

WWF-WA6 Small table made from solid sepetir wood, walnut veneer, and finished engineered wood:

Sepetir is presumed to refer to species of *Sindora*, and walnut to refer to species of *Juglans*, specifically those that are not a part of the butternut group, which is anatomically distinguishable from the core walnuts, which are in turn separable into the tropical black walnuts, the North American walnuts, and European walnut (Miller 1976).

The specimens from this product were identified as: *Eucalyptus* sp. and *Betula* sp., other wood particles that could not be identified, as well as *Hevea* cf. *brasiliensis*. None of these are consistent with the species claim.

WWF-WA7 Hand tool with a mahogany handle:

Mahogany is presumed to be a species of *Swietenia*, unless the name is preceded by some other modifier (e.g. African mahogany, which is presumed to be *Khaya*, or Philippine mahogany, which is presumed to be *Shorea*.) All three accepted species of *Swietenia* are CITES Appendix II species, meaning that any product made from *Swietenia* should reasonably be traceable back to a valid CITES export/import permit. The presence of CITES-controlled species in either Appendix II or Appendix III in a wood product should probably be considered at least a yellow flag. Any CITES Appendix I wood is fully prohibited from trade and is an automatic red flag.

The specimens from this product were identified as: *Carya* sp. This is not consistent with the species claim. *Carya* is a superior choice for a pick handle, as it is famed for its density, strength, and especially its impact resistance.

WWF-WA8 Table made from keruing wood:

Keruing is presumed to refer to any of a number of species of *Dipterocarpus*.

The specimens from this product were identified as: *Dipterocarpus* sp.. This is consistent with the species claim.

WWF-WA9 Seat made from keruing wood:

Keruing is presumed to refer to any of a number of species of *Dipterocarpus*.

The specimens from this product were identified as: *Eucalyptus* sp. This is not consistent with the species claim. *Eucalyptus* and *Dipterocarpus* are neither confusable nor interchangeable for most applications.

WWF-WA10 Kitchen item made from iroko (African teak):

African teak is often synonymous with iroko, but also can refer to such a range of woods as to have no meaning. Fortunately, the claim of iroko is much more precise, and is presumed to refer to one of two species of *Milicia*, which is the current scientific name for the genus formerly known as *Chlorophora*, which was once in turn known as *Maclura*. The two species are *Milicia excelsa* and *Milicia regia*, and they are not separable by wood anatomy.

The specimens from this product were identified as: *Milicia* cf. *excelsa*. This is consistent with the species claim.

WWF-WA11 Meranti wood bench:

Meranti is presumed to refer to species of *Shorea*. Wood anatomically, *Shorea* is identifiable in five essentially distinct groups, the white merantis, the yellow merantis, the light red merantis, the dark red merantis, and the balau group. Common names for these groups notwithstanding, a wood of the balau group would be considered consistent with a general product claim of meranti, as both are still *Shorea*. If the claim were a specific meranti (e.g. yellow meranti) then an identification of any of the other merantis would be inconsistent.

The specimens from this product were identified as: *Shorea* sp., light red meranti group. This is consistent with the species claim.

WWF-WA12 Kitchen item made with solid oak top:

Oak is presumed to be a species of *Quercus*. Wood anatomy recognizes three groups of species within *Quercus*, the red oak group, the white oak group, and the live oak group.

The specimens from this product were identified as: *Quercus*, white oak group. This is consistent with the species claim.

WWF-WA13 Taun solid wood flooring:

Taun is presumed to be a species of *Pometia,* probably *P. pinnatum*.

The specimens from this product were identified as: *Xerospermum* sp., closely related to the claimed species, but once the product claim was known, it was possible to confirm that the specimens was fully consistent with *Pometia*.

WWF-WA14 Solid mahogany table:

Mahogany is presumed to be a species of *Swietenia*, unless the name is preceded by some other modifier (e.g. African mahogany, which is presumed to be *Khaya*, or Philippine mahogany, which is presumed to be *Shorea*.) All three accepted species of *Swietenia* are CITES Appendix II species, meaning that any product made from *Swietenia* should reasonably be traceable back to a valid CITES export/import permit. The presence of CITES-controlled species in either Appendix II or Appendix III in a wood product should probably be considered at least a yellow flag. Any CITES Appendix I wood is fully prohibited from trade and is an automatic red flag.

The specimens from this product were identified as: *Swietenia* sp.. This is consistent with the species claim.

WWF-WA15 Acacia kitchen implement:

Acacia is the common name for the genus *Acacia* (as with *Boa constrictor* or *Tyrannosaurus rex*). It is worth noting that most authors and texts are using the old definition of *Acacia*, which is quite distinct from the modern definition of the genus which is now restricted to predominantly to species from Australia. The iconic *Acacia* of the African savanna are now relegated to the genus *Senegalia*, other species to *Vachellia,* and the former *Acacia* of the new world are mostly in the genera *Mariosousa* and *Acaciella*. In this report, we use the concept of *Acacia sensu lato*. This usage should provide the most generous interpretation of a given product claim with the greatest benefit of the doubt provided to the company. Additionally, the level of botanical confusion surrounding this issue is high, even for botanists, so it is plausible that a responsible effort to engage in due diligence surrounding importing "*Acacia*" that is actually *Vachellia* could result in unintentional and nearly unavoidable 'misrepresentation'. Using the former, broader definition of *Acacia* will minimize this problem.

The specimens from this product were identified as: *Acacia* cf. *confusa*. This is consistent with the species claim.

WWF-WA16 Bubinga instrument:

Bubinga is presumed to include the African species of *Guibourtia*, including *G. demeusii, G. arnoldiana, G. tessmannii,* and *G. ehie.* Three species in the genus are CITES Appendix II species, *G. tessmannii, G. demeusei,* and *G. pellgriniana,* meaning that any product made from one of these species should reasonably be traceable back to a valid CITES export/import permit, however, the CITES and non-CITES species in the genus cannot be separated by wood anatomy. The presence of CITES-controlled species in either Appendix II or Appendix III in a wood product should probably be considered at least a yellow flag even if species level identification is not possible. Any CITES Appendix I wood is fully prohibited from trade and is an automatic red flag.

The specimens from this product were identified as: *Guibourtia* sp. This is consistent with the species claim.

WWF-WA17 Brazilwood violin bow with ebony frog:

Brazilwood is presumed to be *Caesalpinia echinata*, the classically preferred wood for violin bows, and the subject of significant research into wood properties that predict musical quality for this application. *Caesalpinia echinata* is a CITES Appendix II species, meaning that any product made from *Caesalpinia echinata* should reasonably be traceable back to a valid CITES export/import permit. The presence of CITES-controlled species in either Appendix II or Appendix III in a wood product should probably be considered at least a yellow flag. Any CITES Appendix I wood is fully prohibited from trade and is an automatic red flag.

Ebony is presumed to be any species of *Diospyros*. All Madagascar species of *Diospyros* are CITES Appendix II species, meaning that any product made from *Diospyros* should be evaluated to determine the likely origin of the wood, and should it come from Madagascar, it should reasonably be traceable back to a valid CITES export/import permit. The presence of CITES-controlled species in either Appendix II or Appendix III in a wood product should probably be considered at least a yellow flag. Any CITES Appendix I wood is fully prohibited from trade and is an automatic red flag.

The specimens from this product were identified as: Sapotaceae consistent with *Manilkara* for the wood claimed as *Caesalpinia*, and *Dalbergia* cf. *melanoxylon* for the wood claimed as *Diospyros*. Neither of these are consistent with the species claim, and the presence of any *Dalbergia* is likely a CITES violation.

WWF-WA18 Sporting implement made of ayous, zebrano (Zebrawood), and spruce:

Ayous is presumed to be *Triplochiton scleroxylon*, though it may also refer to *Triplochiton zambesicus*, which is not known to be separable by wood anatomy from *T. scleroxylon*.

Zebrano is presumed to be *Microberlinia brazzavillensis*.

Spruce is presumed to be a species of *Picea*, most of which are not separable by wood anatomy.

The specimens from this product were identified as: *Triplochiton* sp., *Microberlinia* sp., and *Paulownia* sp. With the exception of the last wood, these are consistent with the species claim. *Paulownia*, a ring-porous hardwood, is not at all confusable with *Picea*, a softwood with gradual intra-annual transition and thus this product is misrepresented.

WWF-WA19 Table originally claimed to be rosewood, but over the course of the study reported on the website as pine.

Rosewood is presumed to refer to any species of *Dalbergia*, unless the name is preceded by some other modifier (e.g. tiete rosewood, which is presumed to be *Guibourtia chodatiana*). All species of *Dalbergia* are now CITES Appendix II species, meaning that any product made from *Dalbergia* should reasonably be traceable back to a valid CITES export/import permit. The presence of CITES-controlled species in either Appendix II or

Appendix III in a wood product should probably be considered at least a yellow flag. Any CITES Appendix I wood is fully prohibited from trade and is an automatic red flag.

Pine is presumed to refer to any species of *Pinus*, unless the name is preceded by a modifier (e.g. Chilean pine or Norfolk pine – *Araucaria*, or silver pine – *Dacrydium*, etc.) unless that modified refers to one of the identifiable subgroups within *Pinus* (e.g. white pine, red pine, yellow pine, hard pines, soft pines).

The specimens from this product were identified as fibers of: species in the yellow pine group (*Pinus*), in the white/red pine group (*Pinus*), *Quercus*, *Liquidambar,* and possibly *Pseudotsuga*. This is not consistent with either species claim, the original one of *Dalbergia*, nor the later claim of pine.

WWF-WA20 Balau chair with a clarifying claim of acacia:

Balau is presumed to be a species of *Shorea*, specifically a high-density species belonging to the identifiable 'balau group'.

Acacia is the common name for the genus *Acacia* (as with *Boa constrictor* or *Tyrannosaurus rex*). It is worth noting that most authors and texts are using the old definition of *Acacia*, which is quite distinct from the modern definition of the genus which is now restricted to predominantly to species from Australia. The iconic *Acacia* of the African savanna are now relegated to the genus *Senegalia*, other species to *Vachellia,* and the former *Acacia* of the new world are mostly in the genera *Mariosousa* and *Acaciella*. In this report, we use the concept of *Acacia sensu lato*. This usage should provide the most generous interpretation of a given product claim with the greatest benefit of the doubt provided to the company. Additionally, the level of botanical confusion surrounding this issue is high, even for botanists, so it is plausible that a responsible effort to engage in due diligence surrounding importing "*Acacia*" that is actually *Vachellia* could result in unintentional and nearly unavoidable 'misrepresentation'. Using the former, broader definition of *Acacia* will minimize this problem. For this product, we do not understand how to interpret the 'oil' designation, other than potentially as a descriptor of a finish.

The specimens from this product were identified as: *Acacia* cf. *mangium*. This is not consistent with the species claim, and *Acacia* and *Shorea* are not confusable, nor are the properties of *Acacia mangium* comparable to balau.

Because the primary claim is listed as balau, this is treated as misrepresented.

WWF-WA21 Rosewood hand implement:

Rosewood is presumed to refer to any species of *Dalbergia*, unless the name is preceded by some other modifier (e.g. tiete rosewood, which is presumed to be *Guibourtia chodatiana*). All species of *Dalbergia* are now CITES Appendix II species, meaning that any product made from *Dalbergia* should reasonably be traceable back to a valid CITES export/import permit. The presence of CITES-controlled species in either Appendix II or Appendix III in a wood product should probably be considered at least a yellow flag. Any CITES Appendix I wood is fully prohibited from trade and is an automatic red flag.

The specimens from this product were identified as: *Dalbergia* cf. *sissoo*. This is consistent with the species claim, which also indicated "Made in India" which is

further consistent. Nonetheless, the presence of *Dalbergia* could be a CITES violation.

WWF-WA22 Purpleheart and maple sporting implement.

Purpleheart is presumed to be a species of *Peltogyne*.

Canadian maple is presumed to be any species of *Acer* from Canada, but most likely referring to the iconic Canada sugar maple, *Acer saccharum*. Separating *Acer saccharum* of Canadian rather than U.S. origin is not possible by wood anatomy.

The specimens from this product were identified as: *Peltogyne* sp. and *Betula* sp. This is not consistent with the species claim, as *Acer* and *Betula* should not be confused or mixed in trade, despite being superficially similar in appearance.

WWF-WA23 Brazilian Cherry and/or harvest mahogany ceiling fan.

Brazilian cherry is presumed to be any of the species of *Hymenaea*.

Harvest mahogany is presumed to be a species of *Swietenia*. All three accepted species of *Swietenia* are CITES Appendix II species, meaning that any product made from *Swietenia* should reasonably be traceable back to a valid CITES export/import permit. The presence of CITES-controlled species in either Appendix II or Appendix III in a wood product should probably be considered at least a yellow flag. Any CITES Appendix I wood is fully prohibited from trade and is an automatic red flag.

The specimens from this product were identified as: *Populus* sp. This is not consistent with the species claim. *Populus* and *Hymenaea* are quite disparate (density, color, origin, uses) and not at all confusable.

WWF-WA24 Ipe flooring tiles:

Ipe is presumed to be any species of *Handroanthus*. In the past, these woods were known as *Tabebuia* spp., lapacho group, as the genus *Tabebuia* had two groups of species with quite distinct wood anatomy and wood properties. The species producing the ipe-type wood were moved into a new genus, *Handroanthus*, which is consistent with wood anatomy.

The specimens from this product were identified as: *Handroanthus* sp. This is consistent with the species claim.

WWF-WA25 Jatoba and maple veneer board game:

Jatoba is presumed to be any of the species of *Hymenaea*.

Maple is presumed to be a species of *Acer*.

The specimens from this product were identified as: a species in the red oak group (*Quercus*), and a tropical hardwood consistent with *Hymenaea*. This is consistent with the species claim, because the claim of maple is presumed to pertain to the light colored squares on the play surface (which were not submitted for testing), whereas *Quercus* was the backing veneer for the board, and there was no clear claim for that wood.

WWF-WA26 Nyato:

Nyato (or nyatoh) is presumed to be any species of *Palaquium*, but also can refer to *Payena*, *Pouteria*, *Madhuca*, and other Asian Sapotaceae. Because most of these genera are not definitively separable by wood anatomy, further because the family is in the process of near-constant botanical revision, and still further because the trade name nyatoh is not entirely specific, any Sapotaceae consistent with the Asian members of the family are considered correctly specified.

The specimens from this product were identified as: *Palaquium/Payena* sp. This is consistent with the species claim.

WWF-WA27 Particle board, meranti wood, veneer, plywood, and engineered wood table:

Meranti is presumed to refer to species of *Shorea*. Wood anatomically, *Shorea* is identifiable in five essentially distinct groups, the white merantis, the yellow merantis, the light red merantis, the dark red merantis, and the balau group. Common names for these groups notwithstanding, a wood of the balau group would be considered consistent with a general product claim of meranti, as both are still *Shorea*. If the claim were a specific meranti (e.g. yellow meranti) then an identification of any of the other merantis would be inconsistent.

The specimens from this product were identified as: *Shorea* sp. This is consistent with the species claim.

WWF-WA28 Brazilian Teak Cumaru flooring products:

Brazilian teak and cumaru are both understood to be species of *Dipteryx*, especially those species from Brazil. A Central American species in this genus is CITES-controlled; separation of the CITES from the non-CITES species cannot be achieved by wood anatomy alone, and depends in part on information about the origin of the wood. That said, very little Central American *Dipteryx* is known to enter the U.S. market, whereas imports of this genus from Brazil are common.

The specimens from this product were identified as: *Guibourtia* sp. or *Hymenaea* sp. Neither genus is consistent with the species claim, nor are either confusable with *Dipteryx*.

WWF-WA29 Guitar:

29A = spruce (website), cedar (product box)
29B = rosewood (website)
29C = mahogany (website)
29D = sapele (website), linden (product box)
29E = non-claimed species (piece inside guitar body to stabilize neck)":

Spruce is presumed to be a species of *Picea*, most of which are not separable by wood anatomy.

Cedar is nigh to meaningless – it can any of a wide range of species of a number of genera. In the American market cedar (without any modifiers) is understood to be a good-smelling softwood, with modifiers to the common name typically indicating increased specificity.

Rosewood is presumed to refer to any species of *Dalbergia*, unless the name is preceded by some other modifier (e.g. tiete rosewood, which is presumed to be *Guibourtia chodatiana*). All species of

Mahogany is presumed to be a species of *Swietenia*, unless the name is preceded by some other modifier (e.g. African mahogany, which is presumed to be *Khaya*, or Philippine mahogany, which is presumed to be *Shorea*.) All three accepted species of *Swietenia* are CITES Appendix II species, meaning that any product made from *Swietenia* should reasonably be traceable back to a valid CITES export/import permit. The presence of CITES-controlled species in either Appendix II or Appendix III in a wood product should probably be considered at least a yellow flag. Any CITES Appendix I wood is fully prohibited from trade and is an automatic red flag.

Dalbergia are now CITES Appendix II species, meaning that any product made from *Dalbergia* should reasonably be traceable back to a valid CITES export/import permit. The presence of CITES-controlled species in either Appendix II or Appendix III in a wood product should probably be considered at least a yellow flag. Any CITES Appendix I wood is fully prohibited from trade and is an automatic red flag.

Sapele is presumed to be *Entandrophragma cylindricum*, which is fairly reliably separable from other species of *Entandrophragma* and is typically sold as a distinct species.

The specimens from this product were identified as: *Picea* cf. *smithiana* or *Picea* cf. *morrisonicola*, *Dalbergia* cf. *latifolia*, *Entandrophragma* cf. *cylindricum, Canarium schweinfurthii* or *Aucoumea klaineana*, and *Chrysophyllum* sp. The penultimate wood is not consistent with the product claim of mahogany, nor is it reasonably confusable with the claimed species. The final wood is also not on the list, but it was from a portion of the guitar with no specific claim.

WWF-WA30 Abarco flooring product:

Abarco is presumed to be a species of *Cariniana*, often in the literature specifically as *Cariniana pyriformis*.

The specimens from this product were identified as: *Manilkara* sp. This is not consistent with the species claim.

WWF-WA31 Sapele mahogany window treatments:

Sapele is presumed to be *Entandrophragma cylindricum*, which is fairly reliably separable from other species of *Entandrophragma* and is typically sold as a distinct species.

The specimens from this product were identified as: *Khaya* sp., and *Entandrophragma* cf. *cylindricum*, the former of which is not consistent with the species claim.

WWF-WA32 Relaxation item with cumaru wood:

Cumaru is understood to be species of *Dipteryx*, especially those species from Brazil. A Central American species in this genus is CITES-controlled; separation of the CITES from the non-CITES species cannot be achieved by wood anatomy alone, and depends in part

on information about the origin of the wood. That said, very little Central American *Dipteryx* is known to enter the U.S. market, whereas imports of this genus from Brazil are common.

The specimens from this product were identified as: *Dipteryx* sp.. This is consistent with the species claim.

WWF-WA33 Solid ramin and oak

33A (Rung - claimed to be ramin)

33B (Bottom shelf veneer - labeled as oak) :

Ramin is presumed to be a species of *Gonystylus*, all of which are CITES Appendix II species, meaning that any product made from *Gonystylus* should reasonably be traceable back to a valid CITES export/import permit. The presence of CITES-controlled species in either Appendix II or Appendix III in a wood product should probably be considered at least a yellow flag. Any CITES Appendix I wood is fully prohibited from trade and is an automatic red flag.

Oak is presumed to be a species of *Quercus.* Wood anatomy recognizes three groups of species within *Quercus*, the red oak group, the white oak group, and the live oak group.

The specimens from this product were identified as: a species in the yellow pine group (*Pinus*) for the wood claimed as *Gonystylus*, and the oak finish shelf was wood-patterned paper adhered to MDF composed of fibers of *Pinus, Quercus, Tilia*, and possibly *Fagus*. Neither result, especially the pine rung, is consistent with the species claim.

WWF-WA34 North American ash kitchen item:

Ash is presumed to be a species of *Fraxinus.*

The specimens from this product were identified as: *Fraxinus* sp. This is consistent with the species claim.

WWF-WA35 Sporting implement with rosewood insert:

Rosewood is presumed to refer to any species of *Dalbergia*, unless the name is preceded by some other modifier (e.g. tiete rosewood, which is presumed to be *Guibourtia chodatiana*). All species of *Dalbergia* are now CITES Appendix II species, meaning that any product made from *Dalbergia* should reasonably be traceable back to a valid CITES export/import permit. The presence of CITES-controlled species in either Appendix II or Appendix III in a wood product should probably be considered at least a yellow flag. Any CITES Appendix I wood is fully prohibited from trade and is an automatic red flag.

The specimens from this product were identified as: *Hymenaea* sp. or *Guibourtia* sp., more likely the latter. This is not consistent with the species claim, and could constitute a CITES violation.

WWF-WA36 Palisander (Rosewood) kitchen implements:

Rosewood is presumed to refer to any species of *Dalbergia*, unless the name is preceded by some other modifier (e.g. tiete rosewood, which is presumed to be *Guibourtia chodatiana*). All species of *Dalbergia* are now CITES Appendix II species, meaning that

any product made from *Dalbergia* should reasonably be traceable back to a valid CITES export/import permit. The presence of CITES-controlled species in either Appendix II or Appendix III in a wood product should probably be considered at least a yellow flag. Any CITES Appendix I wood is fully prohibited from trade and is an automatic red flag.

The specimens from this product were identified as: *Dalbergia* sp. This is consistent with the species claim, but may still constitute a CITES violation.

WWF-WA37 Sheesham table:

Sheesham (more commonly in the technical literature as shisham) is understood to be one of two species of *Dalbergia,* either *Dalbergia sissoo* or *Dalbergia latifolia.* In the latter case, shisham would usually only be applied to *D. latifolia* of Indian or near-India origin, not from this species in southeast Asia. More commonly, shisham is considered more specific of *D. sissoo*. All species of *Dalbergia* are now CITES Appendix II species, meaning that any product made from *Dalbergia* should reasonably be traceable back to a valid CITES export/import permit. The presence of CITES-controlled species in either Appendix II or Appendix III in a wood product should probably be considered at least a yellow flag. Any CITES Appendix I wood is fully prohibited from trade and is an automatic red flag.

The specimens from this product were identified as: *Dalbergia* cf. *sissoo*. This is consistent with the species claim, but may still constitute a CITES violation.

WWF-WA38 Ramin wood sporting item:

Ramin is presumed to be a species of *Gonystylus*, all of which are CITES Appendix II species, meaning that any product made from *Gonystylus* should reasonably be traceable back to a valid CITES export/import permit. The presence of CITES-controlled species in either Appendix II or Appendix III in a wood product should probably be considered at least a yellow flag. Any CITES Appendix I wood is fully prohibited from trade and is an automatic red flag.

The specimens from this product were identified as species in the red pine group (*Pinus*). This is not consistent with the species claim – *Pinus* and *Gonystylus* are not interchangeable.

WWF-WA39 Oak light switch plate:

Oak is presumed to be a species of *Quercus*. Wood anatomy recognizes three groups of species within *Quercus*, the red oak group, the white oak group, and the live oak group.

The specimens from this product were identified as: a species in the red oak group (*Quercus*). This is consistent with the species claim.

WWF-WA40 Ash light switch plate:

Ash is presumed to be a species of *Fraxinus*.

The specimens from this product were identified as: *Fraxinus* sp. This is consistent with the species claim.

WWF-WA41 Rosewood tray:

Rosewood is presumed to refer to any species of *Dalbergia*, unless the name is preceded by some other modifier (e.g. tiete rosewood, which is presumed to be *Guibourtia chodatiana*). All species of *Dalbergia* are now CITES Appendix II species, meaning that any product made from *Dalbergia* should reasonably be traceable back to a valid CITES export/import permit. The presence of CITES-controlled species in either Appendix II or Appendix III in a wood product should probably be considered at least a yellow flag. Any CITES Appendix I wood is fully prohibited from trade and is an automatic red flag.

The specimens from this product were identified as: *Dalbergia* cf. *sissoo*. This is consistent with the species claim, but may nonetheless constitute a CITES violation.

WWF-WA42 Brazilian teak bench:

Teak (without any modifiers) is presumed to be *Tectona grandis.* Teak occurs natively in southeast Asia, especially Myanmar, but is grown in plantation across much of the tropical world. Growth rate can be used to make broad inferences about the likelihood of plantation origin of teak. That said, Brazilian teak is usually referring to cumaru (*Dipteryx*). It is not clear from the product advertisement whether they mean *Dipteryx* or *Tectona*.

The specimens from this product were identified as: *Tectona* cf. *grandis*. This is consistent with the species claim, given the vagueness of the situation – this is consistency by technicality.

WWF-WA43 Wenge seat:

Wenge is presumed to be any dark, hard, heavy species of *Millettia*, the species of which are not anatomically separable.

The specimens from this product were identified as: *Hevea* cf. *brasiliensis*. This is not consistent with the species claim – *Millettia* and *Hevea* are not at all confusable.

WWF-WA44 Rosewood pen case:

Rosewood is presumed to refer to any species of *Dalbergia*, unless the name is preceded by some other modifier (e.g. tiete rosewood, which is presumed to be *Guibourtia chodatiana*). All species of *Dalbergia* are now CITES Appendix II species, meaning that any product made from *Dalbergia* should reasonably be traceable back to a valid CITES export/import permit. The presence of CITES-controlled species in either Appendix II or Appendix III in a wood product should probably be considered at least a yellow flag. Any CITES Appendix I wood is fully prohibited from trade and is an automatic red flag.

The specimens from this product were identified as: *Lithocarpus* sp. This is not consistent with the species claim, and *Lithocarpus* really bears no semblance to *Dalbergia*.

WWF-WA45 Birch, linden and ramin wood personal beauty item:

Birch is presumed to be a species of *Betula*.

Linden is presumed to be a species of *Tilia*.

Ramin is presumed to be a species of *Gonystylus*, all of which are CITES Appendix II species, meaning that any product made from *Gonystylus* should reasonably be traceable back to a valid CITES export/import permit. The presence of CITES-controlled species in either Appendix II or Appendix III in a wood product should probably be considered at least a yellow flag. Any CITES Appendix I wood is fully prohibited from trade and is an automatic red flag.

The specimens from this product were identified as: *Carpinus* sp. This is not consistent with the species claim, but *Carpinus* is typically found in temperate Eurasian products, when found at all.

WWF-WA46 Birch, linden and ramin wood personal beauty item:

Birch is presumed to be a species of *Betula*.

Linden is presumed to be a species of *Tilia*.

Ramin is presumed to be a species of *Gonystylus*, all of which are CITES Appendix II species, meaning that any product made from *Gonystylus* should reasonably be traceable back to a valid CITES export/import permit. The presence of CITES-controlled species in either Appendix II or Appendix III in a wood product should probably be considered at least a yellow flag. Any CITES Appendix I wood is fully prohibited from trade and is an automatic red flag.

The specimens from this product were identified as: *Carpinus* sp. This is not consistent with the species claim, but *Carpinus* is typically found in temperate Eurasian products, when found at all.

WWF-WA47 Merbau body, mahogany,maple neck, and rosewood fingerboard guitar

47A Fingerboard (rosewood)

47B,C Neck (mahogany & maple)

47D Body (merbau):

Rosewood is presumed to refer to any species of *Dalbergia*, unless the name is preceded by some other modifier (e.g. tiete rosewood, which is presumed to be *Guibourtia chodatiana*). All species of *Dalbergia* are now CITES Appendix II species, meaning that any product made from *Dalbergia* should reasonably be traceable back to a valid CITES export/import permit. The presence of CITES-controlled species in either Appendix II or Appendix III in a wood product should probably be considered at least a yellow flag. Any CITES Appendix I wood is fully prohibited from trade and is an automatic red flag.

Mahogany is presumed to be a species of *Swietenia*, unless the name is preceded by some other modifier (e.g. African mahogany, which is presumed to be *Khaya*, or Philippine mahogany, which is presumed to be *Shorea*.) All three accepted species of *Swietenia* are CITES Appendix II species, meaning that any product made from *Swietenia* should reasonably be traceable back to a valid CITES export/import permit. The presence of CITES-controlled species in either Appendix II or Appendix III in a wood product should probably be considered at least a yellow flag. Any CITES Appendix I wood is fully prohibited from trade and is an automatic red flag.

Maple is presumed to be a species of *Acer*. There is the possibility to separate some maples by wood anatomy, so a more precise common name could yield a more testable claim.

Merbau is presumed to be *Intsia bijuga*.

The specimens from this product were identified as: *Dalbergia, Shorea, Acer, Betula* and *Instia*. This is not consistent with the species claim, because *Shorea* and *Betula* were not in the claim. It is probable that the "mahogany" could have been intended as Philippine mahogany (which is understood to be *Shorea*) but it was not communicated as such.

WWF-WA48 Chinese oak furniture:

Chinese oak is presumed to be a species of *Quercus*, but could also potentially include *Pasania* and *Cyclobalanopsis*. Wood anatomy recognizes three groups of species within *Quercus*, the red oak group, the white oak group, and the live oak group.

The specimens from this product were identified as: *Hevea* cf. *brasiliensis*. This is not consistent with the species claim – *Hevea* and *Quercus* are neither confusable nor interchangeable.

WWF-WA49 Solid wood table with an oak veneer top:

Oak is presumed to be a species of *Quercus*. Wood anatomy recognizes three groups of species within *Quercus*, the red oak group, the white oak group, and the live oak group.

The specimens from this product were identified as: *Hevea* cf. *brasiliensis*, *Populus* sp., and red oak group (*Quercus*) veneer over fiberboard. This is consistent with the species claim, as other than the oak veneer, the only claim was "solid wood".

WWF-WA50 Pine, albasia and meranti woods, particle board, MDF, and veneer furniture:

Pine is presumed to refer to any species of *Pinus*, unless the name is preceded by a modifier (e.g. Chilean pine or Norfolk pine – *Araucaria*, or silver pine – *Dacrydium*, etc.) unless that modified refers to one of the identifiable subgroups within *Pinus* (e.g. white pine, red pine, yellow pine, hard pines, soft pines).

Albasia is presumed to be a misspelling of albizia, one common name for woods of the genus *Albizia*.

Meranti is presumed to refer to species of *Shorea*. Wood anatomically, *Shorea* is identifiable in five essentially distinct groups, the white merantis, the yellow merantis, the light red merantis, the dark red merantis, and the balau group. Common names for these groups notwithstanding, a wood of the balau group would be considered consistent with a general product claim of meranti, as both are still *Shorea*. If the claim were a specific meranti (e.g. yellow meranti) then an identification of any of the other merantis would be inconsistent.

The specimens from this product were identified as: *Albizia* cf. *falcataria* face and back, core veneer cf. *Swietenia* sp., *Mangifera* sp., *Durio* sp., and *Enterolobium* cf. *contortisiliquium*. This is not consistent with the species claim, as the latter three woods were not claimed.

WWF-WA51 Mahogany veneers over mahogany solids furniture:

Mahogany is presumed to be a species of *Swietenia*, unless the name is preceded by some other modifier (e.g. African mahogany, which is presumed to be *Khaya*, or Philippine mahogany, which is presumed to be *Shorea*.) All three accepted species of *Swietenia* are CITES Appendix II species, meaning that any product made from *Swietenia* should reasonably be traceable back to a valid CITES export/import permit. The presence of CITES-controlled species in either Appendix II or Appendix III in a wood product should probably be considered at least a yellow flag. Any CITES Appendix I wood is fully prohibited from trade and is an automatic red flag.

The specimens from this product were identified as: *Swietenia* sp., probably *Khaya* sp., and *Swietenia* cf. *mahagoni* - possibly plantation-grown. These are consistent with the species claim because the 'probable' designation for *Khaya* is not strong enough to assert misrepresentation, and if it were not *Khaya* it would be identified as *Swietenia* based on its microscopic structure.

WWF-WA52 Table with solid Brazilian cherry wood legs, top cherry veneer over MDF:

Brazilian cherry is presumed to be any of the species of *Hymenaea*.

The specimens from this product were identified as: a species in the Lecythidaceae, probably a species of *Cariniana*. This is not consistent with the species claim, nor are *Hymenaea* and *Cariniana* confusable.

WWF-WA53 Balau outdor furniture:

Balau is presumed to be a species of *Shorea*, specifically a high-density species belonging to the identifiable 'balau group'.

The specimens from this product were identified as: *Entandrophragma* sp. This is not consistent with the species claim, and would indicate African origin of the timber rather than southeast Asia.

WWF-WA54 Teak furniture item:

Teak (without any modifiers) is presumed to be *Tectona grandis.* Teak occurs natively in southeast Asia, especially Myanmar, but is grown in plantation across much of the tropical world. Growth rate can be used to make broad inferences about the likelihood of plantation origin of teak.

The specimens from this product were identified as: *Tectona grandis*. This is consistent with the species claim.

WWF-WA55 Solid meranti wood and veneer furniture:

Meranti is presumed to refer to species of *Shorea*. Wood anatomically, *Shorea* is identifiable in five essentially distinct groups, the white merantis, the yellow merantis, the light red merantis, the dark red merantis, and the balau group. Common names for these groups notwithstanding, a wood of the balau group would be considered consistent with a general product claim of meranti, as both are still *Shorea*. If the claim

were a specific meranti (e.g. yellow meranti) then an identification of any of the other merantis would be inconsistent.

The specimens from this product were identified as: *Balfourodendron* sp. This is not consistent with the species claim, and would indicate a South American rather than southeast Asian origin of the timber.

WWF-WA56 Shorea outdoor furniture:

Shorea is presumed to be species of *Shorea*. This would be a more precise synonym for meranti.

The specimens from this product were identified as: a species in the family Anacardiaceae, but unable to narrow it down further. This is not consistent with the species claim, despite the vague identification of the unknown – *Shorea* is a member of the Dipterocarpaceae, not the Anacardiaceae.

WWF-WA57 Iroko mirror:

Iroko is presumed to refer to one of two species of *Milicia*, which is the current scientific name for the genus formerly known as *Chlorophora*, which was once in turn known as *Maclura*. The two species are *Milicia excelsa* and *Milicia regia*, and they are not separable by wood anatomy.

The specimens from this product were identified as: *Milicia* sp.. This is consistent with the species claim.

WWF-WA58 Counter Stool - "solid wood construction" and "grooved American Oak seat”:

Oak is presumed to be a species of *Quercus*. It is not possible by wood anatomy to determine the origin of oak. Wood anatomy recognizes three groups of species within *Quercus*, the red oak group, the white oak group, and the live oak group.

The specimens from this product were identified as: *Hevea* cf. *brasiliensis*. This is not consistent with the species claim, as *Hevea* and *Quercus* are not confusable.

WWF-WA59 Spanish cedar-lined humidor:

Spanish cedar is presumed to be a species of *Cedrela*, most commonly assumed to be *Cedrela odorata*. *C. odorata*, *C. fissilis*, and *C. lilloi* are CITES Appendix III woods, meaning that any product made from one of these three species of *Cedrela* should reasonably be traceable back to a valid CITES export/import permit. The presence of CITES-controlled species in either Appendix II or Appendix III in a wood product should probably be considered at least a yellow flag. Any CITES Appendix I wood is fully prohibited from trade and is an automatic red flag.

The specimens from this product were identified as: very probably *Tectona* sp. veneer quite thin, possibly *Carapa* sp., and *Canarium schweinfurthii* or *Aucoumea klaineana*. Upon further consideration and revisiting the slides and specimens, the features that support an identification of *Tectona* are also consistent with a slightly odd *Cedrela*. As noted it the full data, the identification of *Carapa* is tentative, and the features in such a thin veneer are consistent with

a somewhat atypical but wholly plausible *Cedrela*. This renders this claim provisionally consistent.

WWF-WA60 Sheesham wood tray:

Sheesham (more commonly in the technical literature as shisham) is understood to be one of two species of *Dalbergia,* either *Dalbergia sissoo* or *Dalbergia latifolia.* In the latter case, shisham would usually only be applied to *D. latifolia* of Indian or near-India origin, not from this species in southeast Asia. More commonly, shisham is considered more specific of *D. sissoo*. All species of *Dalbergia* are now CITES Appendix II species, meaning that any product made from *Dalbergia* should reasonably be traceable back to a valid CITES export/import permit. The presence of CITES-controlled species in either Appendix II or Appendix III in a wood product should probably be considered at least a yellow flag. Any CITES Appendix I wood is fully prohibited from trade and is an automatic red flag.

The specimens from this product were identified as: *Dalbergia* cf. *latifolia*. This is consistent with the species claim, or at the least is not inconsistent with the claim, as *Dalbergia latifolia* also grows in southeast Asia, but there is not referred to as shisham.

WWF-WA61 Acacia chair:

Acacia is the common name for the genus *Acacia* (as with *Boa constrictor* or *Tyrannosaurus rex*). It is worth noting that most authors and texts are using the old definition of *Acacia*, which is quite distinct from the modern definition of the genus which is now restricted to predominantly to species from Australia. The iconic *Acacia* of the African savanna are now relegated to the genus *Senegalia*, other species to *Vachellia,* and the former *Acacia* of the new world are mostly in the genera *Mariosousa* and *Acaciella*. In this report, we use the concept of *Acacia sensu lato*. This usage should provide the most generous interpretation of a given product claim with the greatest benefit of the doubt provided to the company. Additionally, the level of botanical confusion surrounding this issue is high, even for botanists, so it is plausible that a responsible effort to engage in due diligence surrounding importing "*Acacia*" that is actually *Vachellia* could result in unintentional and nearly unavoidable 'misrepresentation'. Using the former, broader definition of *Acacia* will minimize this problem.

The specimens from this product were identified as: *Acacia* cf. *mangium*. This is consistent with the species claim.

WWF-WA62 Ash veneer and Asian hardwoods furniture item:

Ash is presumed to be a species of *Fraxinus.*

Asian hardwoods would be any hardwood consistent with an Asian origin (e.g. rubberwood could be considered an Asian hardwood despite being of South American origin, as it is grown in plantations in Asia for natural rubber production and almost exclusively enters the forest products market via the decommissioning of Asian rubber plantation trees).

The specimens from this product were identified as: *Fraxinus* sp., and *Hevea* cf. *brasiliensis*. This is consistent with the species claim, as noted above regarding the likely origin of *Hevea* wood products.

WWF-WA63 Oak hardwoods and birch veneer furniture item:

Oak is presumed to be a species of *Quercus*. Wood anatomy recognizes three groups of species within *Quercus*, the red oak group, the white oak group, and the live oak group. Birch is presumed to be a species of *Betula.*

The specimens from this product were identified as: paper. These specimens were not wood. This is not consistent with the species claim.

WWF-WA64 Zebra wood veneer over Indonesian mahogany solids furniture item:

Zebra wood is presumed to be *Microberlinia brazzavillensis*.

Mahogany is presumed to be a species of *Swietenia*, unless the name is preceded by some other modifier (e.g. African mahogany, which is presumed to be *Khaya*, or Philippine mahogany, which is presumed to be *Shorea*.) All three accepted species of *Swietenia* are CITES Appendix II species, meaning that any product made from *Swietenia* should reasonably be traceable back to a valid CITES export/import permit. The presence of CITES-controlled species in either Appendix II or Appendix III in a wood product should probably be considered at least a yellow flag. Any CITES Appendix I wood is fully prohibited from trade and is an automatic red flag.

The specimens from this product were identified as: *Swietenia* sp., and *Microberlinia* cf. *brazzavillensis* and thus consistent with the species claim, as there are known plantation of *Swietenia* in Indonesia.

WWF-WA65 Solid sheesham wood with MDF back furniture item:

Sheesham (more commonly in the technical literature as shisham) is understood to be one of two species of *Dalbergia,* either *Dalbergia sissoo* or *Dalbergia latifolia.* In the latter case, shisham would usually only be applied to *D. latifolia* of Indian or near-India origin, not from this species in southeast Asia. More commonly, shisham is considered more specific of *D. sissoo*. All species of *Dalbergia* are now CITES Appendix II species, meaning that any product made from *Dalbergia* should reasonably be traceable back to a valid CITES export/import permit. The presence of CITES-controlled species in either Appendix II or Appendix III in a wood product should probably be considered at least a yellow flag. Any CITES Appendix I wood is fully prohibited from trade and is an automatic red flag.

The specimens from this product were identified as: *Dalbergia* cf. *latifolia* and *Acacia* sp. The presence of *Acacia* is not consistent with the species claim.

WWF-WA66 Solid oak with an oak veneer cord panel phone charging station:

Oak is presumed to be a species of *Quercus.* Wood anatomy recognizes three groups of species within *Quercus*, the red oak group, the white oak group, and the live oak group.

The specimens from this product were identified as: a species in the white oak group (*Quercus*). This is consistent with the species claim.

WWF-WA67 Solid rosewood handle outdoor cooking implement:

Rosewood is presumed to refer to any species of *Dalbergia*, unless the name is preceded by some other modifier (e.g. tiete rosewood, which is presumed to be *Guibourtia chodatiana*). All species of *Dalbergia* are now CITES Appendix II species, meaning that any product made from *Dalbergia* should reasonably be traceable back to a valid CITES export/import permit. The presence of CITES-controlled species in either Appendix II or Appendix III in a wood product should probably be considered at least a yellow flag. Any CITES Appendix I wood is fully prohibited from trade and is an automatic red flag.

The specimens from this product were identified as: *Lithocarpus* sp. This is not consistent with the species claim, as *Lithocarpus* is not confusable with *Dalbergia*.

WWF-WA68 Mahogany serving item:

Mahogany is presumed to be a species of *Swietenia*, unless the name is preceded by some other modifier (e.g. African mahogany, which is presumed to be *Khaya*, or Philippine mahogany, which is presumed to be *Shorea*.) All three accepted species of *Swietenia* are CITES Appendix II species, meaning that any product made from *Swietenia* should reasonably be traceable back to a valid CITES export/import permit. The presence of CITES-controlled species in either Appendix II or Appendix III in a wood product should probably be considered at least a yellow flag. Any CITES Appendix I wood is fully prohibited from trade and is an automatic red flag.

The specimens from this product were identified as: *Swietenia* sp. This is consistent with the species claim.

WWF-WA69 Oak frame bulletin board:

Oak is presumed to be a species of *Quercus.* Wood anatomy recognizes three groups of species within *Quercus*, the red oak group, the white oak group, and the live oak group. Cork oak (*Quercus suber*) cannot readily be separated from other members of the white oak group.

The specimens from this product were identified as: paper glued to MDF – not wood. This is not consistent with the species claim.

WWF-WA70 Cumaru flooring product:

Cumaru is understood to be species of *Dipteryx*, especially those species from Brazil. A Central American species in this genus is CITES-controlled; separation of the CITES from the non-CITES species cannot be achieved by wood anatomy alone, and depends in part on information about the origin of the wood. That said, very little Central American *Dipteryx* is known to enter the U.S. market, whereas imports of this genus from Brazil are common.

The specimens from this product were identified as: *Eucalyptus* sp. This is not consistent with the species claim. Most species of *Eucalyptus*, especially those grown in plantations in Brazil where cumaru would have come from, do not have properties to compete with cumaru as a stair nose.

WWF-WA71 Spanish cedar board:

Spanish cedar is presumed to be a species of *Cedrela*, most commonly assumed to be *Cedrela odorata*. *C. odorata*, *C. fissilis*, and *C. lilloi* are CITES Appendix III woods, meaning that any product made from one of these three species of *Cedrela* should reasonably be traceable back to a valid CITES export/import permit. The presence of CITES-controlled species in either Appendix II or Appendix III in a wood product should probably be considered at least a yellow flag. Any CITES Appendix I wood is fully prohibited from trade and is an automatic red flag.

The specimens from this product were identified as: *Cedrela* sp. This is consistent with the species claim, but may well be a CITES violation.

WWF-WA72 Mahogany wood and okoume veneer furniture item:

Mahogany is presumed to be a species of *Swietenia*, unless the name is preceded by some other modifier (e.g. African mahogany, which is presumed to be *Khaya*, or Philippine mahogany, which is presumed to be *Shorea*.) All three accepted species of *Swietenia* are CITES Appendix II species, meaning that any product made from *Swietenia* should reasonably be traceable back to a valid CITES export/import permit. The presence of CITES-controlled species in either Appendix II or Appendix III in a wood product should probably be considered at least a yellow flag. Any CITES Appendix I wood is fully prohibited from trade and is an automatic red flag.

Okume is presumed to be *Aucoumea klaineana.* This is the only species in the genus, but the genus can be confused with other genera in the family.

The specimens from this product were identified as: *Swietenia* sp., *Canarium*/*Daryodes* or *Aucoumea klaineana*, and *Albizia* cf. *falcataria*. The presence of this latter wood is not consistent with the species claim. *Albizia falcataria* is a common shade and nitrogen-fixing species in coffee plantations and is co-planted as an N-fixer in *Eucalyptus* so the presence of this wood may indicate the harvest of over-large or otherwise decommissioned trees in a plantation.

WWF-WA73 Cumaru flooring product:

Cumaru is understood to be species of *Dipteryx*, especially those species from Brazil. A Central American species in this genus is CITES-controlled; separation of the CITES from the non-CITES species cannot be achieved by wood anatomy alone, and depends in part on information about the origin of the wood. That said, very little Central American *Dipteryx* is known to enter the U.S. market, whereas imports of this genus from Brazil are common.

The specimens from this product were identified as: *Diplotropis* sp., and *Eucalyptus* sp. Neither of these woods are consistent with the species claim, nor are they confusable with *Dipteryx*.

Reference:

Miller, R.B. 1976. Wood anatomy and identification of species of *Juglans*. *Botanical Gazette* 137: 368-377.
